# Scaling of ovipositor size in two species of *Drosophila*: altered genital coupling mechanisms and evolution of allometric slopes

**DOI:** 10.1098/rsbl.2025.0295

**Published:** 2025-09-17

**Authors:** Takashi Matsuo

**Affiliations:** ^1^ Agricultural and Environmental Biology, The University of Tokyo, Bunkyo, Tokyo, Japan

**Keywords:** static allometry, negative allometry, the spotted wing *Drosophila*, one-size-fits-all, sexual selection, coevolution

## Abstract

Animal genitalia typically exhibit limited size variation relative to overall body size, a pattern known as negative allometry. The ‘one-size-fits-all’ hypothesis suggests that genital compatibility between sexes constrains the evolution of extreme genital sizes, yet direct evidence remains scarce. *Drosophila suzukii* presents a unique opportunity to test this hypothesis from the female perspective. This species has evolved an enlarged, sclerotized ovipositor capable of piercing intact fruit skins. However, this innovation necessitated an altered genital coupling mechanism, as the modified ovipositor—also functioning as part of the female genitalia—posed a mechanical obstacle to copulation. In contrast, *D. subpulchrella*, the closest relative of *D. suzukii,* retains the ancestral coupling mechanism, which depends on genital size compatibility between sexes. Allometric analyses revealed that *D. subpulchrella* ovipositors exhibit shallower scaling slopes than other body parts, consistent with the negative allometry rule. Conversely, *D. suzukii* ovipositors display significantly steeper slopes, suggesting that the new coupling mechanism has relaxed the constraint on genital size. These findings provide novel support for the one-size-fits-all hypothesis, offering unprecedented resolution into the role of coupling mechanisms in shaping genital allometry.

## 1. Introduction

Static allometry describes the scaling relationship between a morphological trait and overall body size among individuals of the same species. In arthropods, genital traits tend to show reduced variability relative to body size, resulting in shallower slopes compared to other body parts when plotted against body size on log–log axes [1]. This pattern, known as negative allometry, represents the situation where larger individuals have disproportionately small genitalia, and *vice versa*.

The ‘one-size-fits-all’ hypothesis has been proposed to explain this phenomenon, suggesting that genital compatibility between sexes constrains the evolution of extreme genital sizes that lead to reduced reproductive opportunities due to mismatches with the majority of the opposite sex [1,2]. Originally, the theory assumed female choice for male stimulatory ability as the underlying mechanism, but it has since been extended to encompass other contact-based genital functions, such as sperm transfer efficiency and the establishment or stabilization of genital coupling [1,3–6]. Similarly, the selection process maintaining shallow allometry was initially attributed to stabilizing selection favouring smaller genitalia in large individuals and larger genitalia in small individuals, but it was later extended to include size-dependent directional selection, where either too small or too large genitalia hinder successful copulation. In the latter case, for instance, smaller genitalia are favoured in large individuals, while small individuals experience little to no selection pressure on genital size [7].

The prediction of the one-size-fits-all hypothesis has been generally supported by empirical data across arthropod taxa, where negative allometry is more frequently observed in genital structures that are functionally involved in copulation [1–7]. Although numerous supportive cases have been accumulated, the one-size-fits-all hypothesis remains largely theoretical or even conceptual [8]. Several technical difficulties hinder its direct validation. First, measuring selection pressure on genital size is complicated by confounding effects from other traits [9]. For example, in the water strider *Aquarius remiges*, males with larger genitalia were more successful in mating [10], while genital scaling followed negative allometry, seemingly contradicting the one-size-fits-all hypothesis [11]. Such results must be interpreted with caution. Simply comparing fitness across individuals with different genital sizes is insufficient, as body size also influences fitness. Larger males may be more successful in male–male competition or coercive mating. Provided that genital size is correlated with body size, it would appear as if males with larger genitalia were more adaptive. To isolate the effect of genital size, residuals from a regression on body size should be used as an explanatory variable. Importantly, multivariate regression should be avoided in this context, as it introduces multicollinearity when models include tightly correlated variables.

Second, genital allometry studies are heavily male-biased [12]. Female genitalia are often soft and internalized, making precise measurements challenging. As a result, the functional roles of female genital structures during copulation remain poorly understood, leaving genital coupling mechanisms speculative. This lack of information limits our understanding of the selection pressures acting on genital traits and the mechanisms constraining their evolution.

Third, evolutionary allometry—the scaling of homologous traits across species—has received limited attention in this context. Comparing allometric slopes between species experiencing different selection pressures on genital traits could provide novel insights into the one-size-fits-all hypothesis. However, most studies focus on static allometry, comparing different genital traits within species, which reinforces the negative allometry rule but does not directly test the hypothesis.

This may be partially arising from the male bias of genital allometry studies. Male genitalia are often mono-functional, used solely for copulation and not maintained for other functions [13]. Once their role in copulation is lost, male genital structures tend to degenerate, showing body-size-independent variation that appears as errors around the regression curve [14]. This makes it difficult to accurately estimate allometric slopes and compare them across species.

**Figure 1.**
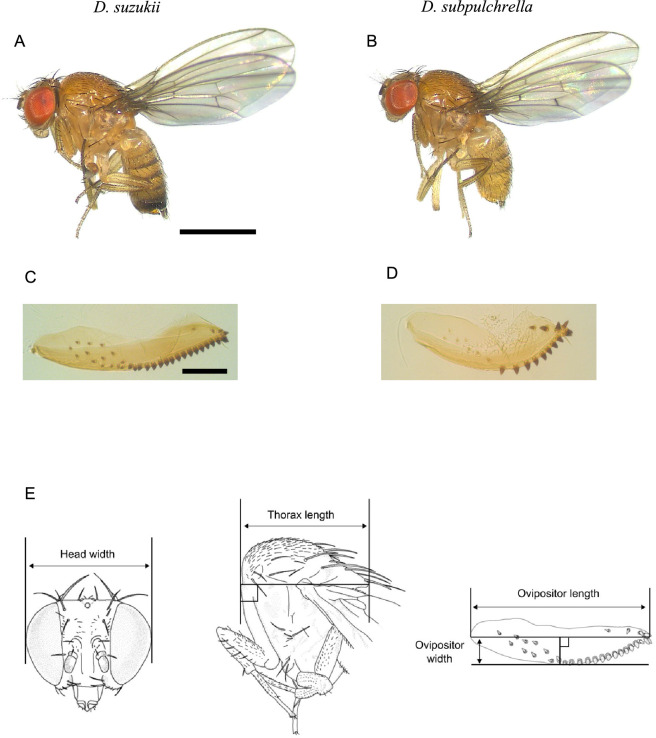
Study system. (A,C) *Drosophila suzukii*. (B,D) *D. subpulchrella*. (A,B) Adult females. Bar = 1 mm. (C,D) Dissected ovipositor. Bar = 100 μm. (E) Definition of traits measured in this study.

*Drosophila suzukii* and *D. subpulchrella* are a pair of closely related species, estimated to have diverged <7.4 million years ago [15]. Both species possess an enlarged, sclerotized ovipositor capable of piercing the skin of fresh fruits, allowing them to exploit a niche unavailable to other *Drosophila* species (figure 1A–D) [16]. *D. suzukii* has a longer and sharper ovipositor than *D. subpulchrella*, enabling it to utilize a broader range of fruits with harder skins, such as cherries and grapes which *D. subpulchrella* cannot use [17]. Due to this capability, *D. suzukii* is considered an agricultural pest worldwide.

In *Drosophila* species, the ovipositor has dual functions. Besides its role in egg laying, it also serves as a female genital structure, facilitating appropriate genital coupling and effective sperm transfer during copulation [18]. *D. subpulchrella* retains the ancestral genital coupling mechanism, where males use a pair of hook-like structures called parameres that contact with the female cuticle near the base of the ovipositor [14]. This mechanism depends on precise size compatibility between the ovipositor and the parameres, as excessively large ovipositors prevent parameres from reaching and anchoring to the appropriate region of the female body. Indeed, surgical disruption of paramere contact shortened copulation duration in *D. subpulchrella*, indicating that parameres play a crucial role in stabilizing genital coupling [14].

In contrast, *D. suzukii* has evolved a novel coupling mechanism in which male parameres no longer contact the female body [14]. The role of parameres stabilizing genital coupling has been replaced by another genital structure called claspers, which grasp the apical end of the ovipositor. This coupling mechanism allows females to have a longer ovipositor without compromising copulation stability. The loss of paramere function has relaxed morphological constraints in *D. suzukii*, as demonstrated by increased paramere shape variation among individuals [14].

**Figure 2.**
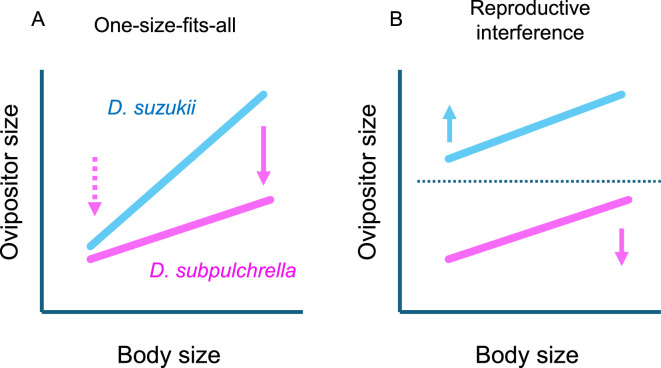
Predictions of alternative hypotheses explaining the mechanism underlying shallow allometric slopes in genital traits. (A) Prediction of the one-size-fits-all hypothesis. In *D. subpulchrella*, the ancestral genital coupling mechanism imposes constraints on absolute ovipositor size, favouring smaller ovipositors in large females but not in small ones. In *D. suzukii*, a novel coupling mechanism has relaxed this constraint, allowing large females to possess larger ovipositors. (B) Prediction of the reproductive interference hypothesis. To prevent interspecific copulation, ovipositor sizes evolve to minimize overlap between the two species. Specifically, selection favours larger ovipositors in small *D. suzukii* females and smaller ovipositors in large *D. subpulchrella* females. As a result, both species exhibit shallow allometric slopes but with distinct intercepts (elevations).

These species provide a rare opportunity to test the one-size-fits-all hypothesis from an evolutionary allometry perspective. In *D. subpulchrella*, the ovipositor size is expected to be under directional and body size-dependent sexual selection, consistent with the assumption of the hypothesis. Its genital coupling mechanism selects against longer ovipositors in large females but not in small ones, provided that the absolute ovipositor size is compatible with the paramere size. Meanwhile, natural selection for the ability to pierce fruit skin would favour larger ovipositors regardless of the body size. Consequently, sexual and natural selections are expected to work antagonistically in large individuals, resulting in a shallow allometric slope for ovipositor size (figure 2A).

In *D. suzukii*, while natural selection remains the same as in *D. subpulchrella*, the constraint arising from genital compatibility is relaxed, allowing large females to have a longer ovipositor. Therefore, we should observe steeper allometric slopes in *D. suzukii* compared to *D. subpulchrella* (figure 2A). It is also expected that the species difference is more pronounced in larger individuals than in smaller ones, because the constraint in *D. subpulchrella* is body size dependent.

In addition to the one-size-fits-all hypothesis, reproductive interference has been considered as an alternative explanation for shallow allometric slopes [2,4]. Reproductive character displacement between closely related species (referred to as the ‘lock-and-key mechanism’ in [2,4]) can reduce interspecies overlap and intraspecies variation in genital morphology, resulting in shallow slopes within each species, accompanied by differences in intercepts (elevation) between species. If ovipositor size differences are crucial for reproductive isolation between *D. suzukii* and *D. subpulchrella*, then small *D. suzukii* females should possess ovipositors larger than those of large *D. subpulchrella* females (figure 2B). Otherwise, large *D. subpulchrella* males potentially copulate with small *D. suzukii* females. Under this scenario, the allometric slope of ovipositor size in *D. suzukii* is expected to be shallow, contrary to the prediction of the one-size-fits-all hypothesis (figure 2). Given that *D. suzukii* and *D. subpulchrella* are often found on the same hosts [19], and interspecies copulation has been observed at least under laboratory conditions [14], reproductive character displacement provides a plausible alternative explanation when shallow slopes are observed in both species.

In this study, the scaling patterns of ovipositors were compared between *D. suzukii* and *D. subpulchrella* to test these alternative explanations for shallow allometric slopes.

## 2. Material and methods

### (a) Sample collection

Two pairs of sympatric populations of *D. suzukii* and *D. subpulchrella*, collected in Tokyo and Yamagata, Japan, were used to assess the consistency of results across populations from different localities. For the Tokyo populations, adults emerged from wild berries (*Rubus hirsutus*, *R. palmatus*, *R. crataegifolius*, *R. microphyllus*, *Cerasus jamasakura*, and *Morus australis*) were directly used for morphological measurements. Ripe fruits were collected from a mountainous area in Hachioji, Tokyo, once a week from May to June in 2024. They were individually maintained in glass vials at 20°C in the laboratory until adults emerged. For the Yamagata populations, laboratory-maintained strains were used for the measurements. These strains were originally established from multiple pairs that emerged from Chinese dogwood fruits (*Cornus kousa*) collected in Minorigaoka, Yamagata, in 2021 [19–21]. They have since been maintained on standard *Drosophila* medium consisting of cornmeal, glucose, and dry yeast at 20°C with a 12 L : 12 D light cycle.

### (b) Morphological measurements

For each female, head width, thorax length, ovipositor length and ovipositor width were measured (figure 1E). The head, thorax and terminal segments of the abdomen were dissected from the rest of the body. Images of the head and the thorax were captured with a digital camera (FLEXACAM C1, Leica Microsystems, Wetzlar, Germany) mounted on a dissection microscope (MZ FL3, Leica Microsystems). The abdominal segments containing the ovipositor was immersed in 10% potassium hydrate overnight to clear the tissues before being mounted on slides with Hoyer’s mountant (Neo-Shigaral, Shiga Insect Company, Tokyo, Japan). The ovipositor consists of a pair of hypogynial valves [22]. They were dissected apart so that both could be measured. Images were captured with a digital camera (FLEXACAM C1) mounted on a phase-contrast microscope (CK X31, Olympus, Tokyo, Japan). To obtain trait sizes in pixel units, the images were analysed using a custom MATLAB script that returns the dimensions of a rectangle drawn around each structure (figure 1E). The same procedure was applied to images of an objective micrometre scale to convert pixel measurements into actual lengths.

### (c) Allometry analysis

For ovipositor length and width, a mean of the two hypogynial valves was used as the representative value for each individual. Differences and correlations between the pairs of valves are shown in the electronic supplementary material, table S1 and figure S1. When either of the hypogynial valves was broken during dissection, the corresponding individuals were excluded from the analysis. Final sample sizes were as follows: Tokyo *D. suzukii*, 42; Tokyo *D. subpulchrella*, 63; Yamagata *D. suzukii*, 70; and Yamagata *D. subpulchrella*, 70. Ordinary least squares (OLS) regression was applied to the log-transformed data using the lm function in R v. 4.3.2 [23]. Because the slopes were compared between species that are likely to have different relative error sizes of the focal trait to that of the body size estimate, OLS was deemed the best method for regression in this study [24]. Scaling of non-genital traits is often not identical even within a species, resulting in different absolute slope values depending on which trait is used as a proxy for body size [9]. To ensure the robustness of the conclusions, two separate analyses were conducted using either head width or thorax length as a body size estimate. The remaining trait was treated as a non-genital reference for slope comparisons. Differences in allometric slopes were tested between species of the same population using the likelihood ratio test (LRT).

## 3. Results and discussion

### (a) Tokyo population

**Figure 3.**
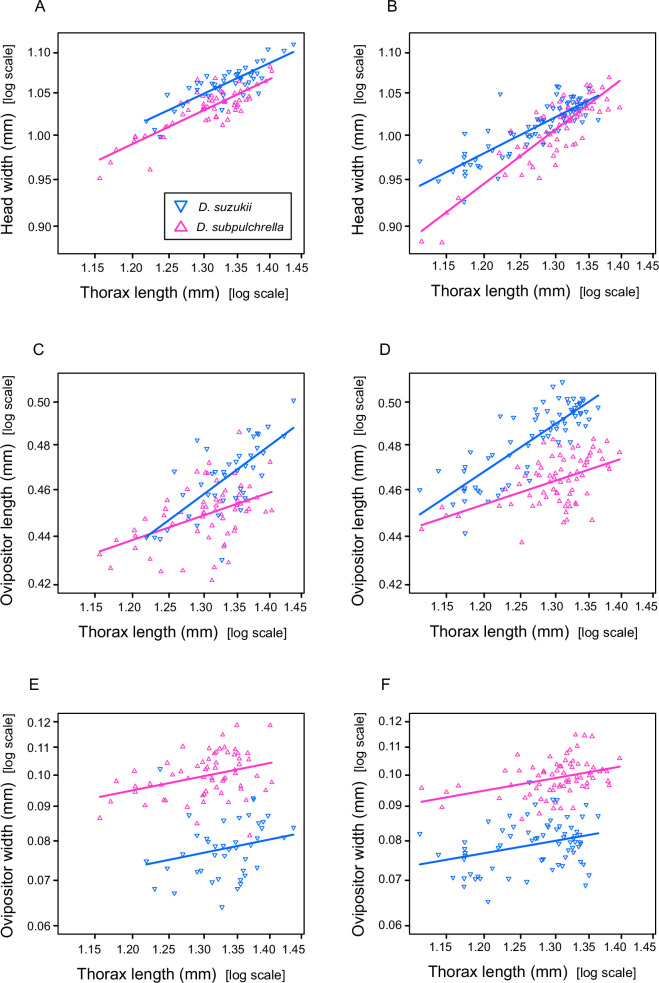
Scaling patterns of head width and ovipositor size against thorax length used as a body size estimate. (A,C,E) Tokyo population. (B,D,F) Yamagata population. (A,B) Head width. (C,D) Ovipositor length. (E,F) Ovipositor width.

When thorax length was used as the body size estimate, the allometric slope of head width did not differ between the two species (table 1, figure 3A; LRT: *F*_1,101_ = 0.014, *p* = 0.907). In both species, the slope values were smaller than 1, showing that head width was less variable than thorax length (table 1; *D. suzukii* 0.48 ± 0.05, *D. subpulchrella* 0.49 ± 0.04).

**Table 1.** Results of allometric analysis.

body size estimate	trait	population	*D. suzukii*		*D. subpulchrella*		slope difference^a^	
			*R* ^2^	slope (s.e.)	*R* ^2^	slope (s.e.)	*F* (d.f.)	*p*-value
thorax length	head width	Tokyo	0.69	0.48 (0.05)	0.70	0.49 (0.04)	0.0138 (1,101)	0.9066
		Yamagata	0.71	0.52 (0.04)	0.75	0.78 (0.06)	15.064 (1,136)	0.0002
	ovipositor length	Tokyo	0.50	0.63 (0.10)	0.21	0.29 (0.07)	7.1883 (1,101)	0.0086
		Yamagata	0.69	0.57 (0.05)	0.26	0.28 (0.06)	15.286 (1,136)	0.0001
	ovipositor width	Tokyo	0.06	0.62 (0.40)	0.12	0.60 (0.21)	0.0008 (1,101)	0.978
		Yamagata	0.10	0.52 (0.19)	0.14	0.53 (0.16)	0.0016 (1,136)	0.9685
head width	thorax length	Tokyo	0.69	1.44 (0.15)	0.70	1.43 (0.12)	0.0024 (1,101)	0.9613
		Yamagata	0.71	1.36 (0.11)	0.75	0.95 (0.07)	10.96 (1,136)	0.0012
	ovipositor length	Tokyo	0.54	1.13 (0.16)	0.27	0.57 (0.12)	7.4313 (1,101)	0.0076
		Yamagata	0.66	0.89 (0.08)	0.32	0.34 (0.06)	31.754 (1,136)	<0.0001
	ovipositor width	Tokyo	0.04	0.87 (0.69)	0.07	0.77 (0.37)	0.0193 (1,101)	0.8899
		Yamagata	0.10	0.84 (0.30)	0.10	0.51 (0.18)	0.987 (1,136)	0.3222

^a^ Probability of sharing the same slope across species was estimated using the likelihood ratio test between two models with a common slope or independent slopes.

In *D. subpulchrella*, the slope for ovipositor length was shallower than that of head width (0.29 ± 0.07 for ovipositor length, 0.49 ± 0.04 for head width; LRT: *F*_1,123_ = 5.31, *p* = 0.023), consistent with the negative allometry rule. In contrast, the slope for ovipositor length was comparable to that for the head width in *D. suzukii* (0.63 ± 0.10 for ovipositor length, 0.48 ± 0.05 for head width; LRT: *F*_1,81_ = 1.76, *p* = 0.189), aligning with the prediction that the constraint on ovipositor size is relaxed in this species. The slopes for ovipositor length were significantly different between the two species (figure 3C, table 1; *F*_1,101_ = 7.19, *p* = 0.009). Notably, the difference was more pronounced in larger individuals (figure 3C), consistent with the expectation that the constraint on ovipositor size in *D. subpulchrella* is body size dependent.

In contrast to ovipositor length, the slopes for ovipositor width were steep in both species and not different from each other (figure 3E, table 1; *D. suzukii* 0.62 ± 0.40, *D. subpulchrella* 0.60 ± 0.21; *F*_1,101_ = 0.0008, *p* = 0.978; note that the *y*-axis range is wider in figure 3E). Although this result was not anticipated, it is consistent with the genital coupling mechanisms in these species, where ovipositor width does not interfere with copulation and is therefore expected to be free from the constraint. The slope values for ovipositor width of both species were comparable to that for *D. suzukii* ovipositor length, showing that ovipositors maintain the same proportion across body sizes in *D. suzukii* but not in *D. subpulchrella*, where the ovipositor becomes tubbier in larger females. Developmental independence between ovipositor length and width highlights the specificity of the constraint on ovipositor length and strengthens support for the one-size-fits-all hypothesis.

These patterns remained consistent when head width was used as the body size estimate, although the absolute slope values increased due to the lower variability of head width compared to thorax length (table 1, bottom half). Slopes for thorax length and ovipositor width did not differ significantly between species (thorax length: *F*_1,101_ = 0.0024, *p* = 0.961; ovipositor width: *F*_1,101_ = 0.0193, *p* = 0.890), whereas the slope for ovipositor length was significantly shallower in *D. subpulchrella* (*D. suzukii* 1.13 ± 0.16, *D. subpulchrella* 0.57 ± 0.12; *F*_1,101_ = 7.43, *p* = 0.008), confirming that the observed interspecific difference is robust and specific to ovipositor length regardless of the trait used as a body size estimate.

### (b) Yamagata population

In contrast to the Tokyo population, the relationship between head width and thorax length was not identical between the two species of the Yamagata population. The allometric slopes for head width and thorax length were significantly different regardless of which trait was used as the body size estimate (table 1, figure 3B; head width: *F*_1,136_ = 15.06, *p* = 0.0002; thorax length: *F*_1,136_ = 10.96, *p* = 0.0012). Comparing the slope values of ovipositor width across all four strains (2 populations × 2 species), it is likely that head width of the *D. subpulchrella* Yamagata strain had an exceptional allometry, resulting in shallower slopes for all the traits in the *D. subpulchrella* Yamagata strain when head width was used as the body size estimate (table 1). This result may reflect geographic variation in scaling of non-genital traits, indicating that head width cannot be used as the body size estimate in the Yamagata population.

Nevertheless, when thorax length was used as the body size estimate, the overall pattern mirrored that of the Tokyo population. The slope for ovipositor length was shallower in *D. subpulchrella* than in *D. suzukii* (table 1, figure 3D; *D. suzukii* 0.57 ± 0.05, *D. subpulchrella* 0.28 ± 0.06; *F*_1,136_ = 15.29, *p* = 0.0001), while the latter was comparable to those of head width and ovipositor width. These results confirm that the morphological constraint on ovipositor length in *D. subpulchrella* is consistent across geographically distinct populations.

### (c) Evolvability of allometric slopes in genitalia

*D. suzukii* has been known to possess a longer ovipositor than *D. subpulchrella*, an adaptation believed to facilitate oviposition in fruits with harder skins. This morphological change could, in theory, have evolved through shifts in allometric intercepts without altering the allometric slope, resulting in uniformly larger ovipositors across body sizes. Indeed, previous studies on the evolvability of genital allometry in other species suggest that intercepts are more evolutionarily labile than slopes. For example, both intra- and interspecies variation in genital allometry among six species of horned beetles was explained mostly by differences in intercepts [25]. Artificial selection experiments demonstrated evolution of intercepts but not slopes in the mosquitofish (*Gambusia holbrooki*) and the guppy (*Poecilia reticulata*) [26,27]. Similarly, ovipositor allometry from independent populations of *D. suzukii* showed variation in intercepts with a consistent slope [21].

In contrast, the present study provides clear evidence for the evolution of allometric slopes between two closely related species. The possibility that these differences arose due to adaptation to laboratory conditions can be ruled out, as the Tokyo population consisted of wild-caught individuals measured directly without laboratory propagation. This finding demonstrates that slope evolution is possible when the constraint imposed by genital compatibility is relaxed, highlighting its role in maintaining shallow slopes in genitalia.

As a result of the slope evolution, the difference between *D. suzukii* and *D. subpulchrella* appeared to be body size dependent. In smaller individuals, ovipositor length was overlapping between the two species, indicating that *D. subpulchrella* males can potentially copulate with small *D. suzukii* females. In other words, ovipositor size alone is insufficient for complete reproductive isolation. This pattern supports the idea that scaling of ovipositor size is influenced by intraspecies genital compatibility rather than by interspecies reproductive interference.

The fitness consequences of genital size incompatibility were not examined in this study. A valuable direction for future research would be to investigate intraspecies variation in allometric intercepts of ovipositor length in *D. subpulchrella*. Such variation may reflect local differences in the equilibrium between ovipositor length and paramere sizes. Inter-population crossing experiments could then be conducted to assess whether females with longer ovipositors experience reduced copulation stability, providing a direct test of the functional impact of genital size incompatibility.

### (d) The one-size-fits-all mechanism as a constraint on ecological adaptation

Muto *et al*. [14] discussed that natural selection favouring longer ovipositors in *D. suzukii* imposed coevolutionary changes in male genitalia, ultimately leading to mechanical reproductive isolation from *D. subpulchrella* [14]. The present findings suggest an alternative scenario: the acquisition of a novel genital mechanism in *D. suzukii* allowed the elongation of the ovipositor, thereby facilitating ecological expansion into harder-skinned fruits. The shallow allometric slope observed in *D. subpulchrella* indicates that the one-size-fits-all mechanism acts antagonistically to natural selection for longer ovipositors, constraining adaptation to harder fruits. In this context, the new genital coupling mechanism in *D. suzukii* may have been a prerequisite for the evolution of longer ovipositors. Without such a shift, individuals with longer ovipositors would have been selected against due to incompatibility with male genital structures. Therefore, release from the constraint imposed by the one-size-fits-all mechanism appears to have been a pivotal event in the ecological adaptation of *D. suzukii*.

### (e) Rarity and generality of this study

The case of *D. suzukii* and *D. subpulchrella* provided a rare opportunity to examine the relationship between genital coupling mechanisms and allometric slopes. Their sclerotized ovipositor not only posed a mechanical obstacle to copulation but also allowed precise morphological measurement. Its dual functions in copulation and egg laying subjected it to antagonizing selection pressures on its size, making it responsive to the altered genital coupling mechanisms. Although such opportunities currently seem limited, deciphering precise genital coupling mechanisms in more species, particularly with an emphasis on female genitalia, could reveal additional cases where allometric slopes are shaped by functional constraints. Accumulating such examples will lead to a better understanding of the mechanisms maintaining shallow slopes in genital allometry.

## 4. Conclusions

This study tested two competing hypotheses to explain the mechanism underlying shallow allometric slopes in genital traits. The scaling patterns of ovipositor length in *D. suzukii* and *D. subpulchrella* supported the one-size-fits-all hypothesis, with *D. suzukii* exhibiting significantly steeper slopes than *D. subpulchrella*. The overlap in ovipositor length between the two species in smaller individuals argues against reproductive interference as the primary mechanism maintaining these scaling patterns. Together, these findings highlight the critical role of genital compatibility between sexes in constraining the evolution of extreme genital sizes.

## Data Availability

The raw data are available from the Dryad Digital Repository [28]. Electronic supplementary material is available online [29].
